# Emergence and clonal spread of colistin resistance due to multiple mutational mechanisms in carbapenemase-producing *Klebsiella pneumoniae* in London

**DOI:** 10.1038/s41598-017-12637-4

**Published:** 2017-10-05

**Authors:** Jonathan A. Otter, Michel Doumith, Frances Davies, Siddharth Mookerjee, Eleonora Dyakova, Mark Gilchrist, Eimear T. Brannigan, Kathleen Bamford, Tracey Galletly, Hugo Donaldson, David M. Aanensen, Matthew J. Ellington, Robert Hill, Jane F. Turton, Katie L. Hopkins, Neil Woodford, Alison Holmes

**Affiliations:** 1 National Institute for Health Research Health Protection Research Unit (NIHR HPRU) in Healthcare Associated Infection and Antimicrobial Resistance at Imperial College London & Public Health England, Hammersmith Hospital, Du Cane Road, W12 0HS London, UK; 2 Imperial College Healthcare NHS Trust, St. Mary’s Hospital, Praed Street, London, W2 1NY UK; 3 Antimicrobial Resistance and Healthcare Associated Infections (AMRHAI) Reference Unit, National Infection Service, Public Health England (PHE), 61 Colindale Avenue, London, NW9 5EQ UK; 4 Department of Infectious Disease Epidemiology, School of Public Health, Imperial College London, London, UK; 5 Centre for Genomic Pathogen Surveillance, Wellcome Genome Campus, Hinxton, Cambridgeshire, UK

## Abstract

Carbapenemase-producing Enterobacteriaceae (CPE) are emerging worldwide, limiting therapeutic options. Mutational and plasmid-mediated mechanisms of colistin resistance have both been reported. The emergence and clonal spread of colistin resistance was analysed in 40 epidemiologically-related NDM-1 carbapenemase producing *Klebsiella pneumoniae* isolates identified during an outbreak in a group of London hospitals. Isolates from July 2014 to October 2015 were tested for colistin susceptibility using agar dilution, and characterised by whole genome sequencing (WGS). Colistin resistance was detected in 25/38 (65.8%) cases for which colistin susceptibility was tested. WGS found that three potential mechanisms of colistin resistance had emerged separately, two due to different mutations in *mgrB*, and one due to a mutation in *phoQ*, with onward transmission of two distinct colistin-resistant variants, resulting in two sub-clones associated with transmission at separate hospitals. A high rate of colistin resistance (66%) emerged over a 10 month period. WGS demonstrated that mutational colistin resistance emerged three times during the outbreak, with transmission of two colistin-resistant variants.

## Introduction

Carbapenemase-producing Enterobacteriaceae (CPE) have emerged globally over the last decade^1,2^. CPE present a ‘triple threat’ of high levels of antibiotic resistance, the ability to cause invasive infections, and spread rapidly^1–3^. A number of centres have reported serious clinical and financial consequences related to the emergence of CPE, including high mortality rates in some reports^3–5^. Rates of CPE are high in some parts of the world (notably parts of Southern Europe, Israel and increasingly parts of the USA)^1,5,6^.

Resistance to carbapenems limits therapeutic options^3,7^. Colistin and other polymyxins have been used for the treatment of CPE^7,8^, but mutational and plasmid-mediated colistin resistance have been reported^7,9–18^. Evidence of horizontal transmission of mobile colistin resistance genes has recently been reported^15,16,18–22^, but few reports have evaluated mutational resistance in a clinical setting coupled with clonal spread^9–11,20,23^.

An outbreak of NDM-producing *Klebsiella pneumoniae* occurred across a multicentre London group of hospitals in 2015, involving patients attending interlinking networks of clinical specialities. We investigated the isolates involved in the outbreak to identify and track the emergence of colistin-resistant CPE using whole genome sequencing.

## Results

### Description of the outbreak

**Figure 1:**
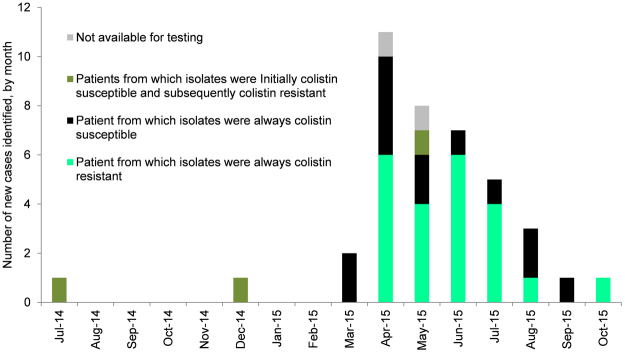
Epidemic curve of the outbreak – incident cases shown. Isolates from three cases were initially colistin susceptible and subsequent isolates from the same cases were colistin resistant.

**Figure 2:**
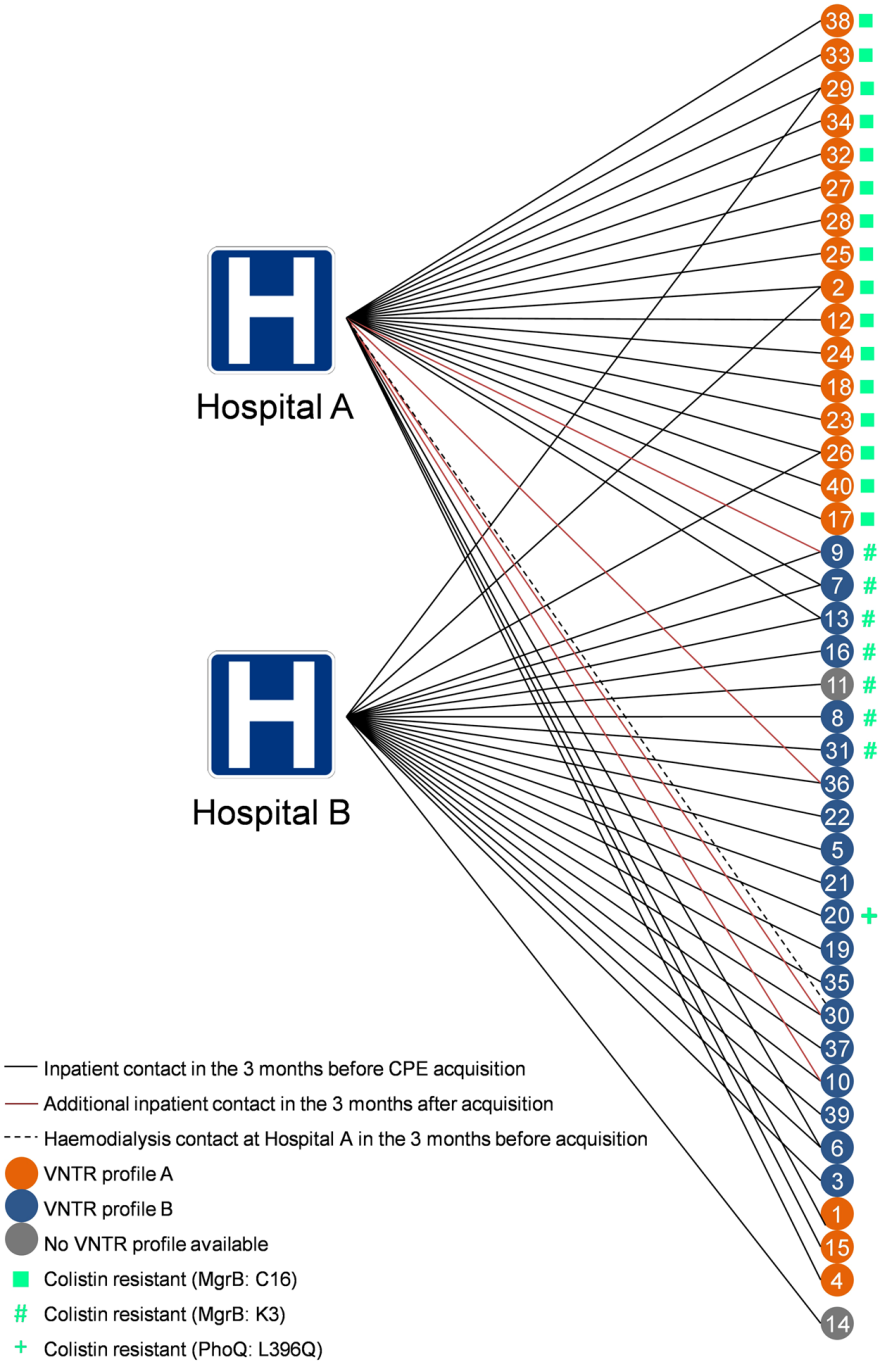
Map of contact of patients with two hospitals in the group. Despite frequent inter-hospital contact, the two VNTR sub-clones remained closely associated with different hospital sites.

A total of 40 patients were identified with the ‘outbreak strains’ between July 2014 and October 2015 (Fig. 1). Twenty-one cases were first identified at Hospital A, mainly from renal inpatient wards, and 19 cases at Hospital B, mainly from vascular inpatient wards. There was frequent contact between the hospitals (Fig. 2).

The clinical characteristics of the patients involved in the outbreak are listed in Table 1. Thirty patients (75%) had antibiotic exposure in the 12 months prior to the initial detection of the outbreak strain (Table 1). No patients had an identified travel history or exposure to healthcare abroad within the preceding 12 months. Twenty-two (55%) patients had a positive clinical specimen at some point during the outbreak period, and 18 (45%) were treated using either colistin or tigecycline. Outcomes were evaluated one year after the outbreak was first identified: of the 40 patients involved in the outbreak, 16 (40%) died and five were discharged with palliative/end of life care plans with no plans for readmission, giving a crude mortality rate of 52%. However, no antibiotic treatment failure-related mortality was identified following detailed clinical review of each of these cases. This may be explained in part by the relatively small number of clinical infections detected during the outbreak (urine 11, skin and soft tissue infection 9, abdominal 4, bloodstream infection 0), and the prompt initiation of combination antimicrobial therapy with colistin and tigecycline in known colonised patients who developed signs and symptoms of an infection, in accordance with local susceptibility patterns. Meropenem was not used as all isolates had an MIC > 32 mg/l. Of the patients surviving the immediate outbreak period, only three were completely discharged from the hospital system. The remainder continue to attend hospital services, including four for regular haemodialysis, and 12 with a variety of inpatient, day case and outpatient attendances.

**Table 1:** Description of the patients involved in the outbreak.

Case number	Age bracket	Speciality at admission	Specimen of first isolate	Subsequent clinical isolate (n days between screen and clinical isolate)^a^	Any antibiotic therapy prior to identification of NDM infection^b^	CPE antibiotic therapy used post NDM identification	Initial colistin MIC (mg/L)^c^	Highest colistin MIC (mg/L)^d^	Colistin resistant at any stage
KP_NDM1	81–90	Renal	Urine	—	Amikacin	No antibiotics	<0.05	**8**	Yes
KP_NDM2	51–60	Vascular	Screening	Liver abscess (5)	Amikacin Metronidazole Piperacillin/Tazobactam Vancomycin	Colistimethate sodium Tigecycline	<0.05	**16**	Yes
KP_NDM3	81–90	Orthopaedic	Urine	—	None identified	No antibiotics	<0.05		
KP_NDM4	61–70	Renal	Abdominal fluid	—	Ciprofloxacin Meropenem Metronidazole Piperacillin/Tazobactam Vancomycin	No antibiotics	<0.05	1	
KP_NDM5	51–60	Vascular	Urine	—	None identified	No antibiotics	<0.05		
KP_NDM6	61–70	Cardiac	Wound (Leg)	—	Meropenem	Colistimethate sodium Tigecycline	Not done	<0.05	
KP_NDM7	71–80	Renal	Screening	Leg swab and tissue, urine (22)	None identified	Colistimethate sodium Tigecycline	**4**	**8**	Yes
KP_NDM8	71–80	Vascular	Screening	None	None identified	No antibiotics	**32**		Yes
KP_NDM9	71–80	Renal	Screening	Urine, central veneous catheter tip (4)	Meropenem	Colistimethate sodium Tigecycline,	**8**		Yes
KP_NDM10	71–80	Renal	Screening	None	Nystatin^a^	No antibiotics	<0.05		
KP_NDM11	81–90	Cardiac	Screening	None	None identified	Colistimethate sodium Tigecycline	**32**		Yes
KP_NDM12	61–70	Renal	Screening and urine	—	Cotrimoxazole^d^	Colistimethate sodium Tigecycline	**8**	**8**	Yes
KP_NDM13	61–70	Cardiac	Screening	None	None identified	No antibiotics	**8**		Yes
KP_NDM14	81–90	Cardiac	Screening	None	Cefalexin	No antibiotics	Not available		
KP_NDM15	61–70	Cardiac	Screening	None	Amikacin Clarithromycin Co–amoxiclav Piperacillin/Tazobactam Vancomycin	No antibiotics	<0.05		
KP_NDM16	81–90	Renal	Screening	Groin wound swab (16)	Meropenem	Tigecycline	**8**		Yes
KP_NDM17	71–80	Renal	Screening	Urine and mouth ulcers (235)	Co–amoxiclav Piperacillin/Tazobactam Vancomycin	Tigecycline	**16**		Yes
KP_NDM18	51–60	Renal	Screening	None	Ciprofloxacin Cotrimoxazole^d^ Metronidazole Piperacillin/Tazobactam, Vancomycin,	No antibiotics	**32**		Yes
KP_NDM19	81–90	Renal	Screening	Urine (6)	Meropenem	Tigecycline	<0.5		
KP_NDM20	71–80	Renal	Screening	Wound swab (11)	None identified	No antibiotics	<0.5	**>32**	Yes
KP_NDM21	51–60	Renal	Screening	Urine (15)	None identified	No antibiotics	1		
KP_NDM22	61–70	Renal	Screening	Urine (254)	None identified	No antibiotics	Not done		
KP_NDM23	61–70	Vascular	Wound (surgical)	—	Ciprofloxacin, Vancomycin	No antibiotics	**32**	**16**	Yes
KP_NDM24	61–70	Cardiac	Screening	None	Co-amoxiclav Ciprofloxacin Vancomycin	No antibiotics	**4**		Yes
KP_NDM25	71–80	Intensive Care	Screening	Wound swabs (groin and thigh) (28)	Metronidazole Piperacillin/Tazobactam Vancomycin	Colistimethate sodium Tigecycline	**4**		Yes
KP_NDM26	61–70	Renal	Screening	None	Piperacillin/Tazobactam, Vancomycin	No antibiotics	**16**		Yes
KP_NDM27	21–30	Intensive Care	Screening	None	Co-trimoxazole^d^ Valganciclovir^d^	No antibiotics	**16**		Yes
KP_NDM28	31–40	Intensive Care	Screening	None	Amikacin Linezolid Liposomal amphotericin,	No antibiotics	**4**		Yes
KP_NDM29	71–80	Vascular	Soft tissue and bone (foot)	—	Amikacin Piperacillin/ Tazobactam, Vancomycin,	No antibiotics	**8**	2	Yes
KP_NDM30	61–70	Cardiac	Screening	None	Ceftriaxone	Colistimethate sodium Tigecycline	1		
KP_NDM31	81–90	Emergency Medicine	Screening	Urine (3)	Ceftriaxone Flucloxacillin Gentamycin, Metronidazole Rifampicin	No antibiotics	**8**		Yes
KP_NDM32	51–60	Maternity	Screening	Urine (18)	Amikacin, Caspofungin Co-trimoxazole^dd^ Ciprofloxacin Metronidazole, Vancomycin	No antibiotics	**8**	**8**	Yes
KP_NDM33	51–60	Intensive Care	Screening	Central veneous catheter tip, abdominal wound (5)	Co-trimoxazole^d^ Meropenem, Nystatin^d^	No antibiotics	**8**		Yes
KP_NDM34	21–30	Renal	Screening	None	Piperacillin/Tazobactam	No antibiotics	**16**		Yes
KP_NDM35	51–60	Renal	Soft tissue and bone (foot)	—	Ciprofloxacin, Clindamycin Vancomycin,	Colistimethate sodium, Tigecycline	<0.5		
KP_NDM36	61–70	Vascular	Screening	None	Anidulanfungin Meropene Rifaximin Vancomycin	No antibiotics	<0.5		
KP_NDM37	91–100	Vascular	Screening	None	Ceftriaxone	No antibiotics	<0.5		
KP_NDM38	0–10	Vascular	Screening	None	Benzylpenicillin Colistimethate sodium, Gentamicin	No antibiotics	**8**		Yes
KP_NDM39	51–60	Vascular	Screening	None	None identified	Colistimethate sodium, Tigecycline	<0.5		
KP_NDM40	61–70	Vascular	Screening	None	Isoniazid^d^	No antibiotics	**8**		Yes

^a^Column denotes patients who were initially identified by screening, but had a subsequent clinical isolate, the site of the first clinical isolate, and the time between the screen and the first clinical isolate.

^b^Identified through case note review/electronic pharmacy records for period 2014/15.

^c^Bold font denotes colistin resistance (>2 mg/L).

^d^Prophylaxis post-transplant as part of approved protocols.

### Colistin resistance

Colistin resistance was detected in isolates from 25/38 (65.8%) patients for which colistin susceptibility was determined; the median colistin MIC was 8 mg/L. Initial isolates from three patients (KP_NDM_1, 2 and 20) were colistin susceptible with a subsequent colistin resistant isolate (Fig. 1). Only 9/25 (36.0%) isolates identified as colistin resistant in the reference laboratory were reported as colistin resistant by the hospital clinical laboratory by disc testing, meaning that colistin resistance was not detected until late in the outbreak in July 2015. Colistin exposure in patients was not significantly associated with having a colistin resistant isolate (10/14 colistin exposed vs. 15/24 not colistin exposed, p = 0.7281). No patients were treated with colistin monotherapy.

Variable number of tandem repeat (VNTR) analysis of *K. pneumoniae* isolates identified two sub-clones, A and B, (Fig. 2); both profiles were indicative of multilocus sequence type ST14. There were two clusters of colistin resistant isolates, one within VNTR sub-clone A on the renal wards in Hospital A, and one within VNTR sub-clone B on the vascular wards at Hospital B (Fig. 2). VNTR sub-clone A was associated with transmission at Hospital A, and sub-clone B with transmission at Hospital B (Fig. 2). All of the 19 patients acquiring VNTR sub-clone A had inpatient contact with Hospital A in the three months prior to acquisition, compared with 4/20 patients who acquired VNTR sub-clone B (p < 0.001), whereas 19/20 patients acquiring sub-clone B had inpatient contact with Hospital B in the three months prior to acquisition, compared with 3/19 patients who acquired sub-clone A (p < 0.001) (Fig. 2).

**Figure 3:**
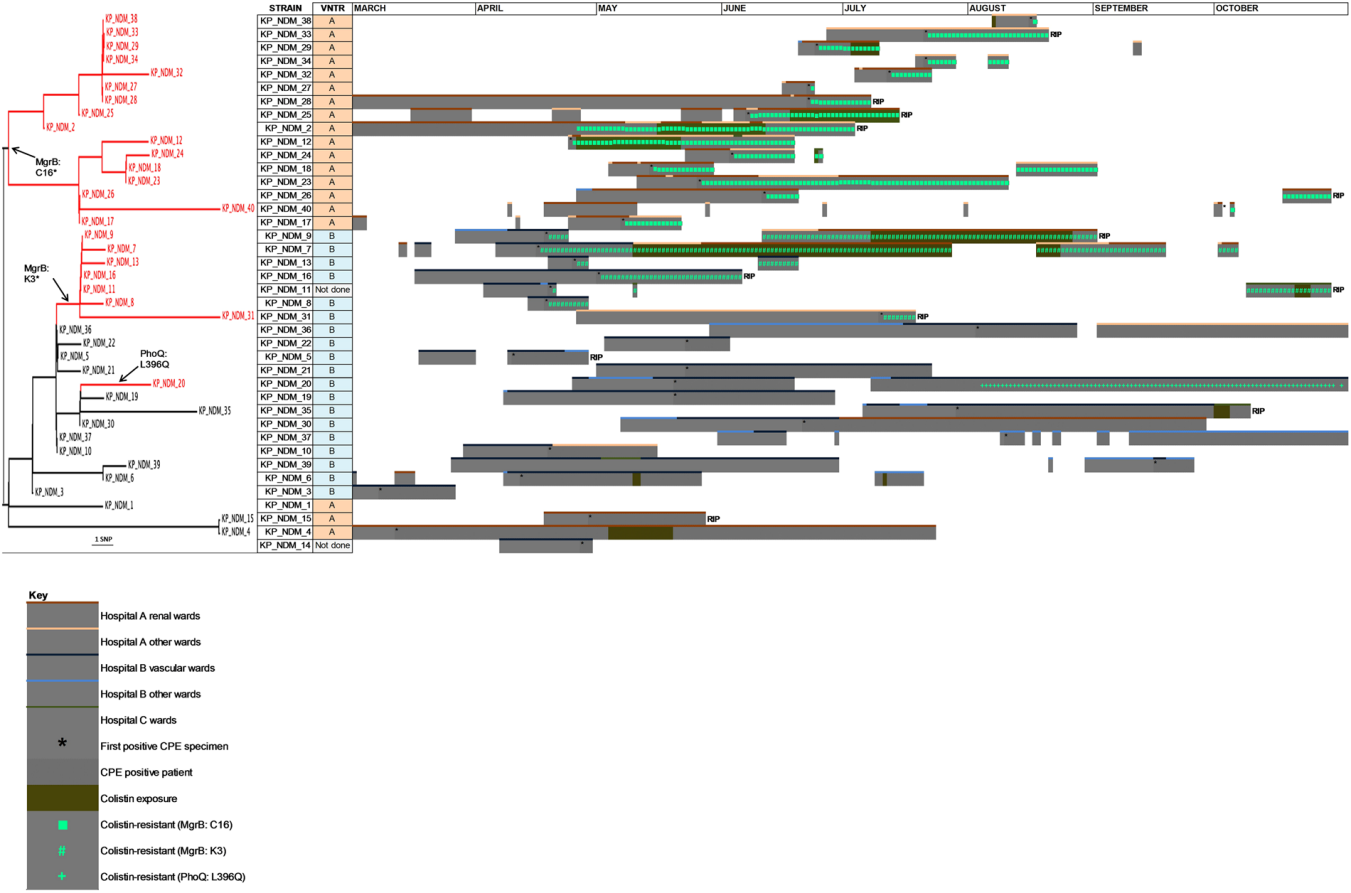
Patient pathways (for 40 patients between March – October 2015) and whole genome sequence Maximum Likelihood tree. The WGS tree supports the emergence and clonal spread of colistin resistance caused by two different mechanisms (MgrB: C16 and MgrB:K3), and also the emergence of a third type of colistin resistance (PhoQ:L396Q) that did not spread clonally. Red colour in tree = colistin resistant isolates; Black colour in tree = colistin susceptible isolates. Isolates KP_NDM_1 and KP_NDM_2 were first identified in July and December 2014, respectively.

WGS showed that the two clusters of colistin resistant isolates were linked with two separate mechanisms of mutational colistin resistance: both due to different mutations each causing an early stop codon in the *mgrB* gene (MgrB: C16 and MgrB: K3) (Fig. 3). A third potential mechanism of mutational colistin resistance, due to a L/Q substitution at amino acid position 396 in *phoQ*, was identified from a single patient (KP_NDM_20), but did not spread clonally (Fig. 3).

The two branches of the dendrogram among the colistin resistant isolates in sub-clone A are consistent with two separate transmission events from the same patient (KP_NDM_2) (Fig. 3). Most of the isolates in the VNTR sub-clone B colistin resistant cluster appeared to have been transmitted on one vascular ward over a short period of time (Fig. 3). A transmission event to a further ward, the ICU, was suggested by the final isolate in this cluster (KP_NDM_31), which occurred on the ICU at Hospital A without obvious epidemiological contact with the vascular wards. The transmission event explaining the identification of this colistin resistant isolate on the ICU most likely occurred around a month earlier during time spent on the same non-renal inpatient ward at Hospital A with patient KP_NDM_7. Interestingly, WGS separated VNTR sub-clone A and B in different phylogenetic branches but with only a few SNPs to distinguish between them, suggesting that they were closely related and may have evolved from a common ancestral strain (Fig. 3).

## Discussion

The therapeutic challenges presented by CPE are exacerbated by the emergence of colistin resistance^7,9–15,17,24,25^. We report a high rate of colistin resistance (66%) caused by three different mechanisms with clonal spread of colistin resistant isolates in two sub-clones circulating in different hospitals during an outbreak of CPE among a network of patients across two hospital sites linked through inpatient stays and dialysis dependence. Colistin resistance was detected late in the outbreak, and highlights the challenges of laboratory detection and therefore the risk for under-ascertainment of colistin resistance^17,20^. Although we did not identify any treatment failure-related mortality, this is in contrast to other studies, and is likely due to the fact that few invasive infections occurred^3,17,26^.

Some Gram-negative bacteria including *Serratia, Brucella* and *Burkholderia* species are inherently resistant to colistin^7^. Acquired colistin resistance can occur through a number of mechanisms: mutational changes in many endogenous genes involved in lipopolysaccharide synthesis, prominently including the *mgrB* gene and upregulation of PhoP/PhoQ, an important two-component sensor-regulator system which impacts lipopolysaccharides^10–14,24,27,28^, and horizontal acquisition of genes as shown by the recent discovery of plasmid-encoding *mcr* genes^15,18–22^. Much attention has focussed recently on plasmid-mediated colistin resistance^15,18–22^. However, our findings suggest that the clonal spread of *K. pneumoniae* with mutational colistin resistance may be a more important clinical threat^9–11,17,20,23,29^. For example, Giani *et al*. report a large hospital outbreak of 93 bloodstream infections caused by KPC-producing *K. pneumoniae* that was mostly explained by clonal expansion of a single *mgrB* deletion mutant^9^. In contrast, in our study, we identified three different mechanisms associated with colistin resistant isolates over the course of a few months. Our findings show that two different types of mutational colistin resistance (both affecting *mgrB*) spread clonally over a short period of time, suggesting that they do not impose a major fitness burden. This underlines the need for robust infection control interventions to prevent the clonal spread of resistance determinants. A mutational change in PhoQ was observed in a single isolate during our outbreak strain, but this variant did not spread to further patients. We were not able to detect the mobile colistin resistance genes *mcr-1*, *-2* or *-3* in any of the study isolates. Understanding of the clinical and epidemiological implications of the various types of colistin resistance is limited, with very little data on the frequency of emergence, fitness impact, and strain variation^7,20,30^. Furthermore, strategies to limit the emergence of colistin resistance (for example optimal colistin dosing and choosing agents that suppress colistin resistance) are in their infancy^7,20,25,31,32^.

The 40 patients involved in the outbreak had complex, extended or repeated, and overlapping inpatient stays and outpatient contact with our hospitals. This made understanding the origin of colistin resistant isolates challenging. Re-ordering epidemiological and patient pathway information based on WGS data provided a useful way to track the emergence and spread of colistin resistance^5,33^. There appeared to be an early division of the outbreak strain into two sub-clones, which circulated concurrently but separately in the renal wards and vascular wards at two hospital sites – and colistin resistance appeared to emerge independently in both sub-clones. Colistin exposure in patients was not associated with colistin-resistant isolates, probably due to the clonal spread of colistin resistant isolates.

Strengths of the study include the combination of epidemiological data and WGS analysis to highlight the emergence and spread of colistin resistant isolates. WGS also facilitated the detection of multiple types of colistin resistance. Although there is an increasing body of evidence linking the mutations identified with colistin resistance^13,14,24,27,28^, limitations of this study include the lack of detailed laboratory and molecular investigations to confirm that the mutations detected are responsible for colistin resistance in these particular isolates, which should be the subject of future studies. The laboratory testing of colistin susceptibility performed locally was not performed using methods recommended by EUCAST or CLSI, which were not available at the time of the outbreak, illustrating the need for timely updates of microbiological testing guidelines. We made inferences about transmission based on WGS phylogeny. This approach is commonly applied in healthcare epidemiology, but the most appropriate sampling strategy(s) and number of SNPs that define a transmission event are not yet clear^5,33^.

Widespread colistin resistance would likely result in increased morbidity, mortality, and direct and indirect costs associated with CPE^3,4,17,26^. Whilst attention has focussed on the plasmid mediated *mcr* genes, the emergence and clonal spread of mutational colistin resistance mediated by three distinct mechanisms over the course of two months during a single outbreak is concerning, and further limits therapeutic options for CPE.

## Methods

### Setting and infection control interventions

The healthcare group comprises five hospitals in London with approximately 1,500 inpatient beds and 190,000 admissions each year. The renal service was located at Hospital A and the vascular service located at Hospital B, with frequent transfer of patients between the two specialties. These hospitals experienced an outbreak of NDM-producing *K. pneumoniae* affecting 40 patients between July 2014 and October 2015^4^. Additional infection control measures launched in April 2015 included enhanced screening (including renal outpatients), contact precautions for known CPE carriers, enhanced chlorine disinfection of the environment, labelling of electronic case notes for identification of readmission, regular teleconference calls internally and with PHE, and enhanced antibiotic stewardship. In response to evidence of ongoing transmission, hydrogen peroxide vapour (HPV) of patient rooms on discharge and ward-based monitors of hand and environmental hygiene were implemented in August 2015.

### Microbiological and molecular investigations

Rectal or faecal screening isolates were plated onto Colorex Supercarba screening agar, and clinical isolates were processed according to local standard operating procedures. Positive colonies were identified by MALDI-TOF and disc susceptibility testing performed according to EUCAST guidelines. Initial colistin susceptibility was recorded as zone present or absent, due to lack of EUCAST interpretative guidelines at the time of the outbreak. Isolates identified as *Klebsiella pneumoniae* that were resistant to ertapenem or meropenem on disc testing had Etest MIC evaluation and PCR performed to screen for carbapenemase genes (Cepheid Xpert® Carba-R).

Isolates confirmed locally as NDM-producing *K. pneumoniae* were referred to Public Health England’s (PHE) Antimicrobial Resistance and Healthcare Associated Infections (AMRHAI) Reference Unit for characterisation. PHE performed whole genome sequencing (WGS) (HiSeq, Illumina, by PHE’s Genomic Services and Development Unit), variable number tandem repeat (VNTR) analysis^34^, and (for 38/40 isolates) MIC determination using an extended panel of antimicrobials (including colistin) by agar dilution. ‘Outbreak strain’ cases were defined as those isolates that were VNTR profile A (6,3,4,0,1,1,1,3,1,1) or B (6,3,4,0,1,1,1,2,1,1); cases were still included if results at some loci were unavailable, provided the other loci matched one of these profiles. Colistin susceptibility was interpreted against EUCAST criteria (R > 2 mg/L).

For WGS analysis, reads from each genome were mapped onto the reference genome (*K. pneumoniae* MGH 78578) using BWA (version 0.7.9a).The SAM file generated thereby was converted to BAM with Samtools (version 1.1). Single nucleotide polymorphisms (SNPs) were called using the Genome Analysis Toolkit 2 (GATK2) and then filtered based on the depth of coverage (DP ≥ 5), ratio of unfiltered reads that support the reported allele compared to the reference (AD ≥ 0.8) and mapping quality (MQ ≥ 30). SNPs filtered out using these metrics, including heterozygotes were designated by ‘N’. SNPs from each genome were thereafter combined to generate a single multiple alignment file with the maximum proportion of Ns accepted at any position of the alignment set to less than 20%. The maximum likelihood (ML) tree was constructed using RAxML. BLAST was used to search for the mobile colistin resistance genes, *mcr-1*, *-2* and *-*3, sequence (using 80% identity) in VelvetOptimiser de novo sequence assemblies.

### Clinical epidemiology

Patients were identified from clinical isolates or screening cultures in accordance with UK national guidelines^35,36^. Patient details were collected prospectively using a data collection form, including patient demographics, diagnosis and comorbidities (ICD10 codes), antimicrobial treatments, and medical interventions. In order to track the emergence and spread of colistin resistant isolates, inpatient pathways of the patients involved in the outbreak were mapped to highlight overlapping inpatient stays and review possible transmission routes. The topology of the ML phylogenetic tree (reconstructed from the WGS data) was used to re-order and plot the patient pathways in order to understand likely transmission routes. Categorical variables were analysed by Fisher’s Exact tests.

This work was classified as service evaluation and exempt from NHS Research Ethics Committee review.

The datasets generated during and/or analysed during the current study are available from the corresponding author.
